# Risky Riding Violations Among Motorcycle Riders Through an Integrated Model of Behavioral, Psychological and Workload Factors

**DOI:** 10.3390/bs16101860

**Published:** 2026-10-09

**Authors:** Pawinee Iamtrakul, Sararad Chayphong

**Affiliations:** Center of Excellence in Urban Mobility and Innovation, Faculty of Architecture and Planning, Thammasat University, Pathumthani 12120, Thailand

**Keywords:** integration model, motorcycle rider, risk behavior, road safety, work condition

## Abstract

Risky violation behavior among motorcycle riders engaged in goods-delivery and service activities poses a significant challenge to road safety, particularly amid the growing demand for platform-based commercial motorcycle services. This research developed a unified structural model to investigate how Theory of Planned Behavior (TPB) constructs, risk perception, personal traits and workload are associated with risky riding violations across behavioral, perceptual, individual and occupational dimensions. A survey of 1400 commercial riders working in food-delivery and ride-hailing services was conducted, with 1251 samples analyzed after screening. Thereafter, the hypothesized associations among the constructs were evaluated through structural equation modeling (SEM). The results suggested that perceived social pressure and personality characteristics were positively associated with risky violation behavior, whereas attitudes and workload demonstrated negative associations with risky violation behavior. Furthermore, when the structural model was examined separately according to work experience, differences in the patterns and strengths of the relationships among the factors were observed. Overall, risky riding violations appear to arise from a combination of behavioral, individual and occupational conditions with the strength and direction of these associations differing across levels of work experience. Therefore, road safety interventions should consider the specific characteristics and experience levels of commercial riders rather than applying a uniform approach to all riders. Tailored strategies addressing the distinct behavioral and contextual factors of different rider groups may contribute to more effective risk reduction and road safety promotion.

## 1. Introduction

Motorcycle riding for goods and service delivery is one of the forms of transportation associated with a high risk of road traffic crashes. This is partly due to the distinctive characteristics of delivery riding, which differ from those of conventional motorcycle riding on public roads. Delivery riding is performed as part of an occupation and is often accompanied by tight delivery deadlines, algorithmic piece-rate pay, long working hours exceeding 8 h per day and urban traffic congestion. These working conditions have been associated with risky riding behaviors among food delivery riders. Strict delivery deadlines and time pressure may encourage aggressive and unsafe riding practices, with time pressure found to be positively associated with aggressive riding behavior among food delivery riders (Dong et al., 2021; Papakostopoulos & Nathanael, 2021). Occupational demands and job-related stress have also been associated with risky riding and lower road-safety compliance among delivery riders (Nguyen-Phuoc et al., 2022; Chen, 2023). Specific violations reported in previous research include speeding, red-light running, riding against traffic flow, and other unsafe or aggressive riding practices (Shen et al., 2020; Dong et al., 2021). These findings indicate that risky riding among delivery riders encompasses multiple forms of traffic violations and unsafe riding practices, highlighting the importance of examining risky violation behavior within the specific occupational context of motorcycle delivery work.

Nevertheless, previous research has examined risky behaviors among general motorcyclists, with comparatively little emphasis on commercial riders. The unique working conditions and job demands experienced by commercial riders warrant closer investigation into the determinants of risky riding violations within this occupational group. Therefore, identifying the factors associated with violation behaviors among motorcycle delivery riders is important for developing a better understanding of their safety risks and informing appropriate road safety interventions. Previous studies investigating the factors influencing risky riding behaviors have frequently adopted the TPB, a widely used theoretical framework for explaining and predicting risky behaviors through behavioral intentions (Ali et al., 2025; Leong et al., 2021). However, recent research has highlighted the limitations of relying solely on the TPB framework. Consequently, a growing body of research has expanded the theoretical framework by incorporating other relevant concepts or theories according to the specific objectives and contexts of each study (Shen et al., 2020; Qian et al., 2024). In the present studies, the importance of expanding the theoretical framework is recognized in addressing the research gap concerning risky riding behaviors among motorcycle riders engaged in goods and service delivery. This gap is particularly important because commercial riders operate under occupational conditions that differ from those of general motorcycle riders, while also being influenced by individual and behavioral factors. However, it remains unclear how these different dimensions are associated with risky violation behavior when considered within the same analytical framework. This raises the central research question of which behavioral, individual, and work-related factors are associated with risky violation behavior among motorcycle delivery riders, and how their relationships differ within an integrated model. Accordingly, this research develops an integrated conceptual framework comprising three dimensions: Theory of Planned Behavior (TPB), individual factors, and work conditions. Specifically, the framework examines the relationships of attitude, subjective norms, perceived behavioral control, risk perception, personal traits and workload with risky violation behavior. Unlike previous studies that have typically incorporated risk perception, personal traits, or work-related factors as separate extensions of behavioral models, this research examines these factors simultaneously within a single integrated framework. By doing so, the research seeks to provide a more comprehensive understanding of the factors associated with risky violation behavior in the specific occupational context of motorcycle delivery riding.

## 2. Literature Review

### 2.1. TPB and Commercial Riders’ Risky Violation Behavior

Behavioral research has been widely applied across various fields and has also been extensively used in traffic psychology research (Chen, 2009). In the context of risky driving behavior, TPB has been applied to many previous studies to explain and predict behavioral intentions and subsequent behaviors, including speeding behavior (Ali et al., 2025; Chen & Chen, 2011), helmet use behavior (Rezapur-Shahkolai et al., 2022), wrong-way driving (Satiennam et al., 2023) and distracted driving (Hoang et al., 2025). Building on the Theory of Reasoned Action (Ajzen & Fishbein, 1980), the TPB proposes that behavioral intentions are shaped by attitudes, subjective norms and perceived behavioral control (Ajzen, 1991). Within this framework, attitude reflects an individual’s evaluation of a behavior based on its expected outcomes, whereas subjective norm represents the perceived social expectations regarding whether a behavior should be performed. Perceived behavioral control reflects how capable individuals believe they are of carrying out a particular action, given the conditions that may enable or hinder it. Thus, a main assumption of the TPB is that intention is the primary factor of behavior (Ajzen, 1988, 1991). Nevertheless, previous studies have also suggested that the three TPB constructs may have direct relationships with actual behavior, indicating that their influence may extend beyond behavioral intention. Despite the extensive application of the TPB in traffic psychology, much of the previous research focused on general risky driving behaviors. In contrast, evidence concerning risky riding behavior among motorcycle riders engaged in delivery and service activities remains relatively limited. This group may experience distinctive behavioral and work-related conditions that differ from those of ordinary motorcycle users. Therefore, examining the relationships between the core TPB constructs and risky violation behavior among commercial riders may provide further insight into how behavioral, social and perceived control factors contribute to risky riding practices. The proposed hypotheses from the existing literature are as follows:

**H1.**  *Attitude has a negative effect on risky violation behavior.*

**H2.**  *Subjective norm has a positive effect on risky violation behavior.*

**H3.**  *Perceived behavioral control has a positive effect on risky violation behavior.*

### 2.2. Individual Factors: Risk Perception and Personal Traits

Although TPB provides a theoretical basis for understanding behavioral determinants, risky riding may also be associated with individual psychological characteristics. In this study, the individual dimension incorporates risk perception and personal traits, which represent riders’ assessments of riding risks and their relatively stable individual characteristics, respectively.

#### 2.2.1. Risk Perception

Risk perception refers to an individual’s assessment of the likelihood and potential consequences of hazardous situations. In the context of motorcycle riding, differences in perceived risk may influence whether riders regard behaviors such as speeding, weaving through traffic, or riding while fatigued as acceptable or unsafe. He et al. (2021) found that risk perception was associated with riding behavior, including an indirect relationship with violations and a direct relationship with riding errors. Vuong et al. (2023) further demonstrated relationships among personality characteristics, riding self-confidence, attitudes, risk perception, and risky riding behavior. These findings indicate that risk perception provides an additional psychological perspective for understanding risky riding beyond the core TPB constructs. For motorcycle delivery riders, risk perception is particularly relevant because riders may repeatedly encounter hazardous situations while simultaneously responding to occupational demands. The way riders perceive the risks and potential consequences associated with these situations may therefore be related to their engagement in risky riding violations. Based on this evidence, the following hypothesis is proposed:

**H4.**  *Risk perception has a negative effect on risky violation behavior.*

#### 2.2.2. Personal Traits

Personality represents another individual characteristic associated with driving and riding behavior. Established personality frameworks include the Revised NEO Personality Inventory (Costa & McCrae, 1992). Among these personality dimensions, neuroticism is particularly relevant to the present study because it reflects characteristics such as emotional instability, anxiety, and sensitivity to negative emotions (McCrae & John, 1992). Previous research has identified relationships between personality characteristics and risky road-user behavior. Higher extraversion and neuroticism, together with lower conscientiousness, have been associated with risky driving and accident-related outcomes (White & Dahlen, 2001). Neuroticism has also been positively associated with aggressive driving (Zhang et al., 2017), while extraversion and neuroticism have been related to unsafe driving behavior (Riendeau et al., 2018). Research further indicates that neuroticism and related emotional characteristics may influence decision-making under stressful conditions and broader everyday functioning (Widiger & Oltmanns, 2017). In the motorcycle context, Vuong et al. (2023) also identified relationships between personality traits and risky riding through psychological mechanisms including attitudes and risk perception. Taken together, these findings indicate that individual personality characteristics may contribute to differences in responses to risky riding situations. This may be particularly relevant among delivery riders who regularly operate under demanding traffic and occupational conditions. Accordingly, the following hypothesis is proposed:

**H5.**  *Personal traits have a positive effect on risky violation behavior.*

### 2.3. Work Conditions: Workload

Workload represents the demands placed on individuals when performing work-related tasks and can be considered from both objective and perceived perspectives. In the context of motorcycle riding, perceived workload reflects riders’ subjective assessment of the demands experienced while performing riding tasks (He et al., 2023). A widely established approach for assessing perceived workload is the NASA Task Load Index (NASA-TLX), developed by Hart and Staveland (1988), which assesses workload through multiple dimensions, including mental demand, physical demand, temporal demand, effort, performance, and frustration. Previous research has examined workload specifically in relation to risky riding behavior. He et al. (2021) found that workload had a positive direct effect on riding violations, although its relative contribution was small. He et al. (2023) further reported that perceived workload directly affected risky driving behavior, while no significant relationship was identified between perceived workload and emotional factors. Together, these findings indicate that perceived workload may have a direct relationship with risky riding behavior and provide the basis for examining workload as a distinct work-condition factor in the present study. Motorcycle delivery riders may experience substantial workload while simultaneously managing riding tasks and delivery responsibilities. Although related occupational factors such as time pressure, job stress, and fatigue have also been investigated in delivery-rider research (Papakostopoulos & Nathanael, 2021; Nguyen-Phuoc et al., 2022; Chen, 2023), these constructs are conceptually distinct from workload and are therefore not incorporated into the workload construct examined in the present study. Accordingly, this study specifically examines perceived workload and proposes the following hypothesis:

**H6.**  *Workload has a positive effect on risky violation behavior.*

### 2.4. Conceptual Dimensions of Risky Riding Behavior

Based on the literature review of risky riding behavior, the factors associated with such behavior can be synthesized into three main dimensions: Theory of Planned Behavior (TPB), individual, and work-condition dimensions. Each dimension comprises distinct latent factors that capture different behavioral, psychological, and occupational aspects of risky riding. The TPB dimension consists of attitude, subjective norms, and perceived behavioral control (PBC); the individual dimension comprises risk perception and personal traits; and the work-condition dimension focuses on workload (see Table 1).

## 3. Materials and Methods

### 3.1. Participants

Participants were categorized by commercial riding experience to examine whether the relationships among behavioral, psychological, and workload factors varied according to experience. A five-year work-experience threshold was selected to reflect the major transition in Thailand’s platform-based transport and delivery sector around 2020–2021. This period coincided with the rapid expansion of platform-based services during the COVID-19 pandemic and was followed by the introduction of the Ministerial Regulation on Ride-Hailing Service Vehicles via an Electronic System B.E. 2564 (2021), an important regulatory milestone for app-based passenger transport in Thailand. Although the regulation applied specifically to passenger cars rather than app-based motorcycle delivery riders, it represents an important point in the development and formal recognition of platform-based transport services in Thailand. Accordingly, the five-year threshold provides a contextual basis for distinguishing riders whose commercial riding experience largely preceded this transition from those who entered the sector more recently. The final analytical sample comprised 1251 riders and was used for the CFA and SEM analyses. Of these, 799 riders had five years or less of commercial riding experience, while 452 had more than five years of experience. Table 2 presents the socio-economic and work characteristics of the overall sample and the two experience groups. The overall sample had a mean age of 43.5 ± 10.5 years and an average driving experience of 17.2 ± 9.6 years. Riders reported an average of 10.7 ± 2.8 driving hours and 24.1 ± 11.0 trips per day, with a mean monthly riding income of 13,588 ± 6581 baht. Riders with five years or more of experience were significantly older (46.4 ± 9.8 vs. 41.9 ± 10.6 years, *p* < 0.001), had greater overall driving experience (20.0 ± 9.7 vs. 15.6 ± 9.2 years, *p* < 0.001), and completed more trips per day (25.7 ± 11.9 vs. 23.2 ± 10.3, *p* < 0.001). No significant differences were observed in household size, daily driving hours, longest continuous work period, or monthly riding income (*p* > 0.05). These group differences were considered when interpreting the experience-stratified analyses.

### 3.2. Study Area and Data Collection

The research was conducted in Bangkok and the neighboring province of Pathum Thani, which was selected as the principal suburban study area. The target population comprised commercial riders providing food delivery and passenger-transport services through both platform-based and traditional service models. The proposed model included 22 variables. Based on the criterion of 20 observations per variable, a minimum sample of 440 respondents was required. Initially, 1400 riders participated in the questionnaire survey. During data screening, 149 responses were excluded because they contained incomplete responses or missing data for variables required in the analysis. Consequently, 1251 complete and valid responses were retained, representing a retention rate of 90%. The final sample therefore exceeded the minimum sample-size requirement and was considered adequate for the statistical analyses. A structured questionnaire was developed around the constructs and relationships represented in the conceptual model. Trained surveyors administered the questionnaire in person at selected locations where eligible riders commonly gathered or operated. Prior to data collection, the surveyors were briefed on participant eligibility, questionnaire administration procedures, and the use of consistent instructions to ensure that the same administration principles were applied across participants. The same questionnaire structure and sequence of items were used for all respondents. The indicators were therefore administered in a predetermined order and were not counterbalanced. Participants were recruited purposively because inclusion in the study depended on meeting the predefined occupational criteria. Ethical clearance was granted by the Human Research Ethics Committee of Thammasat University, Faculty of Social Sciences (Approval No. 072/2025). Before participation, respondents received information about the study’s objectives, their right to participate voluntarily and the measures used to protect their identity and personal information. Informed consent was obtained from every participant.

### 3.3. Measurement and Data Analysis

The study comprised 7 latent variables and 22 observed variables. The independent variables included four main factors. First, the TPB constructs consisted of attitude, measured by two observed indicators (α = 0.695); subjective norm, measured by three observed indicators (α = 0.734), and perceived behavioral control, measured by three observed indicators (α = 0.743). All items were measured using a five-point Likert scale based on the level of agreement, ranging from 1 = strongly disagree to 5 = strongly agree, except for the workload indicators. Second, workload was measured using four indicators (α = 0.802) adapted from the NASA-TLX framework, which is widely used to assess perceived workload across occupational settings (Hart & Staveland, 1988). Four dimensions were included: mental demand, temporal demand, effort and frustration, each rated on a scale ranging from 1 = low to 20 = high. The standard NASA-TLX pairwise-weighting procedure was not applied; instead, the four unweighted dimension scores were used directly as indicators of the workload construct in the SEM. Third, risk perception was measured using four observed indicators (α = 0.772). The items were assessed by using a five-point Likert scale based on the level of agreement, ranging from 1 = strongly disagree to 5 = strongly agree. Fourth, personal traits were measured using three observed indicators (α = 0.814), representing tendencies related to being easily upset, becoming angry easily and being easily irritated. The items were assessed using a five-point Likert scale ranging from 1 = strongly disagree to 5 = strongly agree. The dependent variable was risky violation behavior, which was measured using three observed indicators (α = 0.757). These indicators represented violation-related riding behaviors, including riding on sidewalks, riding against traffic and speeding. The items were assessed using a five-point frequency scale ranging from 1 (never) to 5 (always).

**Figure 1:**
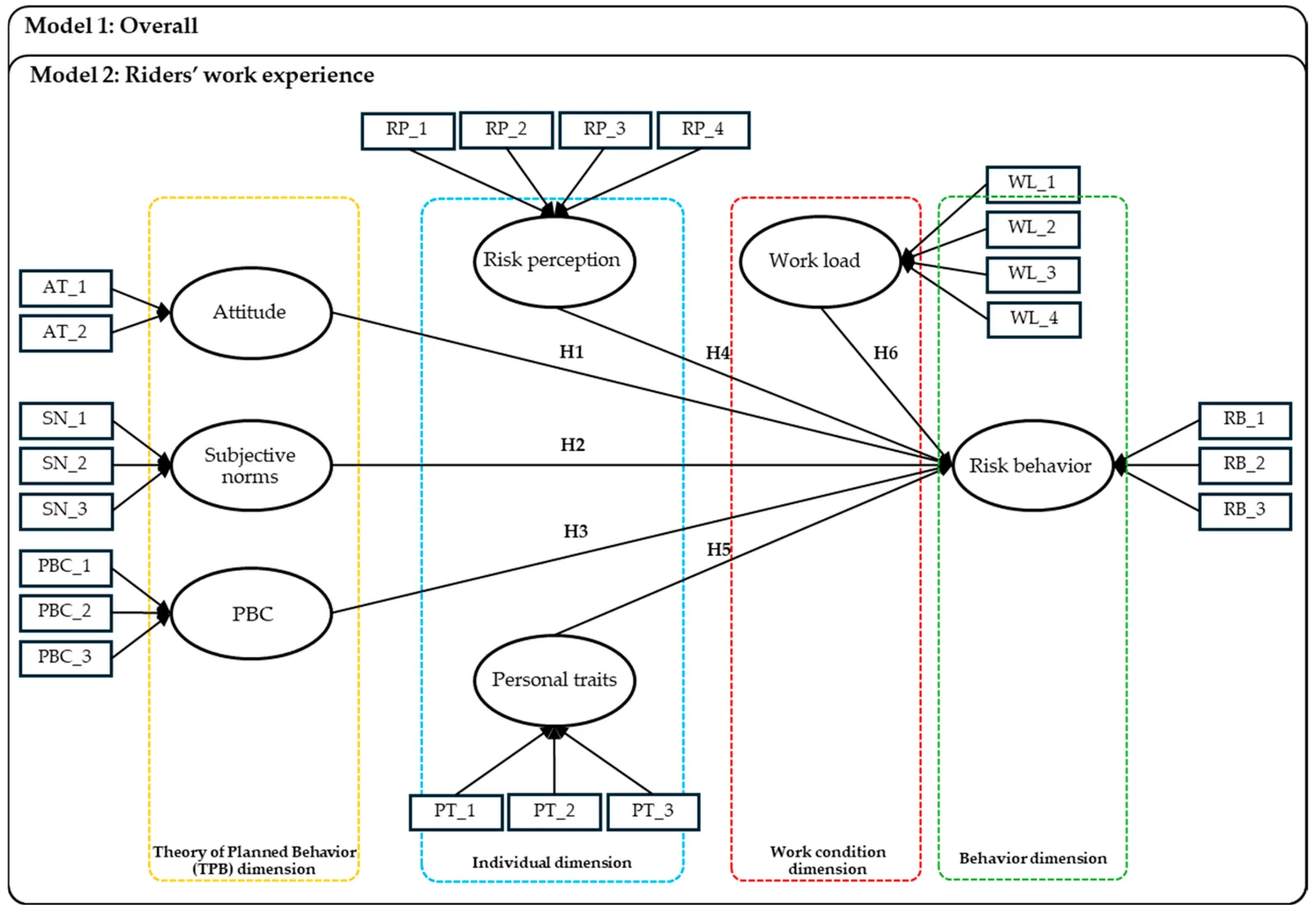
Hypothesized Structural Equation Model.

The analytical framework employed Confirmatory Factor Analysis (CFA) and Structural Equation Modeling (SEM). CFA was first used to assess the measurement model and determine whether the observed indicators adequately represented the latent constructs: attitude, subjective norm, perceived behavioral control, risk perception, personal traits, workload and risky violation behavior. SEM was subsequently used to test the hypothesized structural relationships between the six explanatory constructs and risky violation behavior (see Figure 1). Specifically, the model tested the relationships of attitude (H1), subjective norm (H2), perceived behavioral control (H3), risk perception (H4), personal traits (H5) and workload (H6) with risky violation behavior. Model 1 tested all six relationships using the full sample. Models 2.1 and 2.2 tested the same structural relationships separately by work experience, with Model 2.1 comprising riders with fewer than five years of work experience and Model 2.2 comprising riders with more than five years of work experience. For each model, standardized path coefficients (β) and their statistical significance were used to evaluate the hypothesized relationships. Model fit was assessed using CMIN/df, SRMR, RMSEA, CFI, TLI, and GFI (Hair et al., 2009).

## 4. Results

### 4.1. General Information of Respondents

The socioeconomic characteristics of the sample indicate that more than 92% of the participants were male, while females accounted for only 8%. The participants had a mean age of 43.54 years (SD = 10.52). More than 97% had an educational level below a bachelor’s degree. In terms of income, more than 80% reported a monthly income between 10,001 and 25,000 THB, while only approximately 5% reported earning more than 25,000 THB per month. Furthermore, as shown in Table 3, the descriptive statistics were examined alongside comparisons of the mean scores of the study variables between riders with different levels of work experience. The results showed statistically significant differences in perceived behavioral control, risk perception, personal traits and workload, indicating that the mean scores of these factors differed significantly between the two groups of riders based on their work experience.

### 4.2. Measurement Model

From Table 4, Model 1, the skewness values of the factors ranged from −0.611 to 1.407, while the kurtosis values ranged from −0.891 to 1.010. For Model 2.1, the skewness values ranged from −0.638 to 1.248, and the kurtosis values ranged from −0.833 to 0.560. For Model 2.2, the skewness values ranged from −0.584 to 1.294, while the kurtosis values ranged from −0.858 to 0.691. These results indicate that the distributions of the factors did not exhibit substantial deviations from normality, with the skewness and kurtosis values remaining within acceptable ranges for SEM analysis (Brown, 2006). For the results of the measurement model quality assessment for the overall model (Model 1) indicated that the standardized factor loadings of all indicators ranged from 0.649 to 0.806, which were within an acceptable range. This indicates that each indicator adequately reflected its corresponding latent construct.

In addition, the Average Variance Extracted (AVE) values for the constructs ranged from 0.461 to 0.596, the Composite Reliability (CR) values ranged from 0.701 to 0.816, and Cronbach’s alpha values ranged from 0.695 to 0.814 (Fornell & Larcker, 1981). Although the AVE and Cronbach’s alpha values for some constructs were slightly below the recommended thresholds, when considered together with the factor loadings and CR values, the results indicate that the measurement scales demonstrated acceptable convergent validity and reliability (Abdi & Williams, 2010; Hair et al., 2009). For the models based on work experience (Models 2.1–2.2), the standardized factor loadings of all indicators ranged from 0.567 to 0.854, which were within an acceptable range. This indicates that each indicator adequately reflected its corresponding latent construct. In addition, AVE values for the constructs ranged from 0.408 to 0.604, the CR values ranged from 0.665 to 0.822, and Cronbach’s alpha values ranged from 0.668 to 0.816. Although the AVE and Cronbach’s alpha values for some constructs were slightly below the recommended thresholds, when considered together with the factor loadings and CR values, the results indicate that the measurement scales demonstrated acceptable convergent validity and reliability (Abdi & Williams, 2010; Hair et al., 2009).

Construct distinctiveness across the models was evaluated using the Heterotrait–Monotrait ratio (HTMT). The HTMT estimates for all construct pairs were below the recommended threshold of 0.85 (Henseler et al., 2015). The highest HTMT estimate was observed between personal characteristics and subjective norms (HTMT = 0.522), indicating adequate differentiation between the constructs (see Table 5). In addition, none of the bootstrapped confidence intervals included 1.00, further supporting the discriminant validity of the constructs across the measurement models.

The CFA results indicated demonstrated an acceptable fit to the empirical data. For the overall model (Model 1), the χ^2^/df was 3.742, with CFI = 0.939, TLI = 0.925, GFI = 0.952, RMSEA = 0.047 and SRMR = 0.042. For Model 2.1, the χ^2^/df was 3.248, with CFI = 0.922, TLI = 0.904, GFI = 0.937, RMSEA = 0.053, and SRMR = 0.047. In contrast, Model 2.2 had a χ^2^/df of 2.813, CFI = 0.893, TLI = 0.868, GFI = 0.904, RMSEA = 0.063 and SRMR = 0.055. Overall, Model 1 and Model 2.1 met the recommended criteria for an acceptable model fit (Hair et al., 2009). For Model 2.2, the CFI and TLI values were slightly below the conventional threshold of 0.90 but remained above 0.80, while the other fit indices were within acceptable ranges (Le et al., 2023).

### 4.3. SEM Result

**Figure 2:**
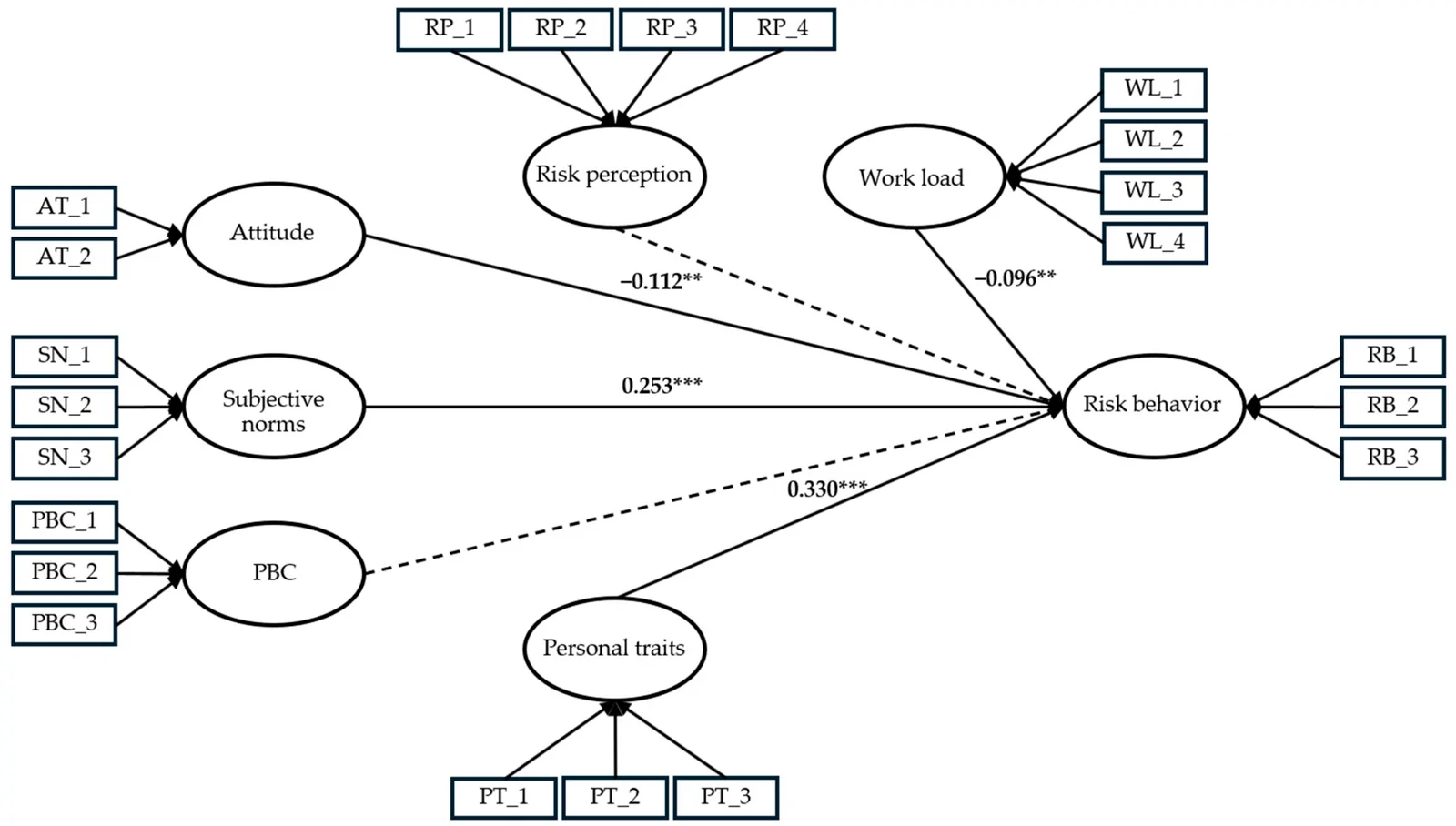
SEM Result for Model 1. Remarks: *** indicates *p* ≤ 0.001, ** indicates *p* ≤ 0.01.

**Figure 3:**
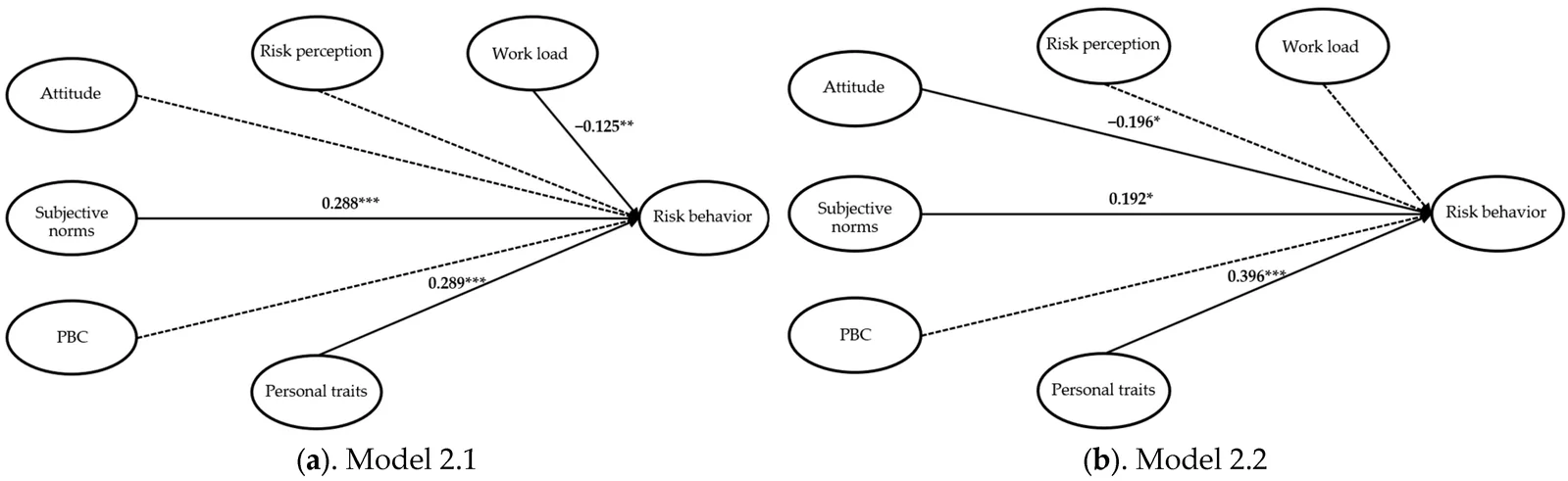
SEM Result for Model 2. Remarks: *** indicates *p* ≤ 0.001, ** indicates *p* ≤ 0.01 and * indicates *p* ≤ 0.05.

For the full sample (Model 1), the SEM estimates showed negative pathways from attitude (β = −0.112) and workload (β = −0.096) to risky violation behavior (see Figure 2). Positive pathways were identified for subjective norms (β = 0.253) and personal traits (β = 0.330). In Model 2.1, workload remained a significant negative predictor (β = −0.125), while subjective norms and personal traits were significant positive predictors, each with a coefficient of 0.288 and 0.289, respectively (see Figure 3a). Model 2.2 produced a different pattern: attitude was negatively associated with risky violation behavior (β = −0.196), whereas subjective norms (β = 0.192) and personal traits (β = 0.396) showed significant positive associations (see Figure 3b).

Overall, in Model 1, H1, H2 and H5 were supported, while H6 was not supported, and H3 and H4 were not statistically significant. The experience-stratified results showed some variation: attitude was significant only among riders with more than five years of experience (Model 2.2), whereas workload was significant only among riders with less than five years of experience (Model 2.1). Notably, workload showed a significant negative relationship with risky violation behavior in Model 1, contrary to the positive relationship proposed in H6 (see Table 6).

## 5. Discussion

### 5.1. TPB Constructs and Risky Violation Behavior

The results indicate that the TPB constructs were associated with risky violation behavior in different ways. Attitude had a significant negative relationship with risky violation behavior, supporting H1. This suggests that riders with stronger safety-oriented attitudes were less likely to report traffic violations. This finding is consistent with previous motorcycle-specific TPB research, in which attitude was an important predictor of risky riding and speeding intentions (Elliott, 2010; Tunnicliff et al., 2012). While these studies primarily examined behavioral intentions, the present study further identifies a direct association between attitude and self-reported risky violation behavior among commercial riders. Subjective norm had a significant positive relationship with risky violation behavior, supporting H2 and indicating the relevance of perceived social expectations to commercial riders’ behavior. Previous studies have similarly identified social influences in motorcycle riding, although their effects vary according to the type of normative influence examined. Elliott (2010) highlighted the relatively limited role of conventional subjective norms compared with broader forms of social influence, while Tunnicliff et al. (2012) distinguished subjective norms from group norms reflecting what other riders do.

Therefore, the present finding should be interpreted as evidence of perceived social expectations, rather than direct evidence of peer normalization. In contrast, perceived behavioral control was not significantly associated with risky violation behavior; therefore, H3 was not supported. Previous evidence regarding PBC has also varied. Elliott (2010) reported mixed findings across motorcycle studies, while Tunnicliff et al. (2012) found PBC to be more consistently related to safe-riding intentions than risky intentions. This suggests that the contribution of perceived control may depend on the specific behavior and outcome examined. Overall, H1 and H2 were supported, whereas H3 was not supported, indicating that attitude and subjective norms had more evident relationships with risky violation behavior than perceived behavioral control in the present model.

### 5.2. Personal Traits and Workload

Among the additional factors incorporated into the model, both personality traits and workload were significantly associated with risky violation behavior, although the two factors showed different patterns of influence. Neuroticism was positively associated with risky riding violations, suggesting that riders with greater emotional instability tended to report more frequent violations. This finding is generally consistent with previous research examining the relationship between personality traits and risky riding or driving behavior. For example, Le et al. (2023) extended the TPB by incorporating personality traits and found that these traits influenced risky behavior among young riders both directly and indirectly through mediating factors. Similarly, Riendeau et al. (2018) found that neuroticism was significantly associated with simulated driving performance. The observed positive association suggests that riders with stronger neurotic tendencies, including emotional instability, may be more prone to committing risky riding violations. One possible explanation is that greater emotional reactivity may influence how riders respond to demanding or stressful situations on the road, potentially affecting their behavioral choices and decision-making. This result strengthens the importance of taking individual personality characteristics into account when examining risky violation behavior among commercial riders, particularly alongside behavioral and work-related conditions. In contrast, workload had a statistically significant negative association with risky violation behavior, indicating that riders with higher perceived workloads reported fewer risky riding behaviors. As this direction was opposite to that proposed in H6, the hypothesis was not supported. This finding differs from He et al. (2021), who reported a positive effect of workload on riding violations and suggested that excessive workload may increase risk-taking. The negative association observed here may reflect the occupational and economic characteristics of the sample. For riders with relatively low incomes who depend on platform-based work, accidents, traffic penalties, or vehicle damage can reduce working time and earnings. Riders facing greater workloads may therefore adopt more cautious riding practices to maintain work continuity and protect their earning capacity. Greater workload may also reflect accumulated riding experience and familiarity with traffic and route conditions. However, this finding should not be interpreted as indicating that higher workload improves safety as excessive workload may still contribute to fatigue and other adverse outcomes.

Therefore, the negative association identified in the present study may reflect characteristics specific to motorcycle delivery and service riders. Rather than indicating that higher workload inevitably leads to greater risky behavior, the findings suggest that riders may respond to increased work demands differently depending on their working conditions, perceived risks, and behavioral adaptations. This highlights the importance of considering the occupational context and characteristics of different rider groups when examining the relationship between workload and risky violation behavior.

### 5.3. Differences by Work Experience

The model was also examined across work-experience groups by comparing riders with five years or less of experience with those who had more than five years of experience. For both groups, subjective norms and personal traits showed significant positive relationships with risky violation behavior. However, different patterns emerged for attitude and workload. Among less-experienced riders, attitude was not significantly associated with risky violation behavior, whereas higher workload was significantly associated with fewer violations. In contrast, among more-experienced riders, attitude showed a significant negative relationship with risky violation behavior, while workload was not statistically significant. These patterns suggest that the factors associated with risky violation behavior may vary according to riders’ work experience. Previous research similarly suggests that riding experience may be associated with differences in motorcycle safety outcomes, although these relationships are not consistent across all behavioral outcomes. For example, Sunmud et al. (2024) identified differences in accident involvement across riding-experience groups but found no corresponding differences in traffic violations. Thus, rather than suggesting that greater experience necessarily leads to safer riding, work experience may represent an important contextual characteristic shaping the relationships between behavioral and occupational factors and riding outcomes. More experienced riders may develop greater familiarity with work routines and strategies for managing occupational demands; however, these mechanisms were not directly examined in the present study and should be regarded as possible explanations rather than established effects. Furthermore, because differences between the two models were not directly tested statistically, variations in coefficient magnitude or significance should not be interpreted as evidence of a moderating effect of work experience. Nevertheless, the observed patterns underscore the importance of considering work experience when examining factors associated with risky violation behavior among motorcycle delivery riders.

### 5.4. Limitation and Future Study

This research has several limitations. First, the research focused on behavioral factors by extending the TPB with additional factors representing individual characteristics and work-related conditions. Future research could further expand the framework by incorporating context-specific factors, such as characteristics of the travel environment, to provide a more holistic understanding of risky violation behavior. Second, work experience was measured based on the duration of employment. However, behavioral patterns may also vary across different operational contexts. Future studies could therefore examine the model across additional contextual dimensions, such as spatial settings (urban, suburban, and rural areas) and working-time conditions (daytime versus nighttime), to determine whether the relationships among the factors vary across different contexts. Third, participants were purposively recruited from Bangkok and Pathum Thani. Therefore, the sample should not be considered statistically representative of commercial riders across Thailand, and the findings may not fully reflect the experiences of riders in other regions. Future research should include more geographically diverse samples to determine whether the observed relationships remain consistent across commercial rider populations or vary according to regional contexts and perceptions. Fourth, questionnaire indicators were administered in the same predetermined order for all participants without counterbalancing. Although this approach ensured consistency in questionnaire administration, potential order and fatigue effects cannot be entirely excluded. Future studies could randomize or counterbalance the order of measures to minimize these potential biases. Overall, future research should incorporate broader contextual factors, more diverse rider populations, and alternative questionnaire administration procedures to provide a more comprehensive understanding of risky violation behavior among commercial riders.

## 6. Conclusions

This research aimed to investigate the associations between behavioral, perceptual, personal and work-related factors associated with risky violation behavior by integrating the TPB, risk perception, personal traits and workload into an integrated structural model. The results highlight the continuing relevance of the TPB-related constructs in explaining risky violation behavior, even when behavioral intention was not incorporated as a mediating mechanism. Among the TPB-related factors, subjective norms emerged as a particularly prominent predictor. When the TPB constructs were considered alongside the extended factors, personal traits demonstrated a strong influence within the overall model. One possible explanation lies in the attributes assessed by the personality measures, especially a heightened tendency to respond negatively through anger, irritability or emotional distress. Such emotional tendencies may contribute to the likelihood of engaging in violation-related riding behaviors, particularly under challenging or stressful situations. Further analysis using separate structural models according to work experience revealed that the patterns and relative strengths of the relationships varied across experience groups. This finding suggests that the mechanisms underlying risky violation behavior may not be uniform among commercial riders but may instead reflect differences in their work experience and individual characteristics.

Accordingly, road safety policies, commercial rider regulations and targeted interventions should account for differences across rider groups while addressing the behavioral, personal and occupational dimensions of rider safety. These may include psychological approaches that promote safety-oriented attitudes and decision-making, the development of emotional regulation and coping skills and improvements in working conditions, particularly in terms of physical demands, time pressure and perceived economic returns. Such targeted and multidimensional approaches may contribute to more effective strategies for reducing risky violation behavior and promoting safer practices among commercial riders.

## Figures and Tables

**Table 1:** Dimensions, Variables and Indicators of Risky Behavior.

Main Variables		Descriptions	References
Theory of Planned Behavior (TPB) dimension			
Attitude	AT_1	Evaluation of risky riding as wrong/unacceptable	Ajzen (1991); Chen (2009); Vuong et al. (2023)
	AT_2	Priority given to safe riding rather than risky/speed-oriented riding	Ajzen (1991); Chen (2009); Chen and Chen (2011); Vuong et al. (2023)
Subjective norms	SN_1	Influence of other riders/peers on risky riding	Ajzen (1991)
	SN_2	Influence of significant others, including friends/family	Ajzen (1991)
	SN_3	External/organizational social influence on risky riding	Ajzen (1991)
PBC	PBC_1	Perceived riding skill and capability	Ajzen (1991); Vuong et al. (2023)
	PBC_2	Perceived ability to control risky riding and its consequences	Ajzen (1991); Vuong et al. (2023)
	PBC_3	Riding self-confidence under risky situations	Ajzen (1991); Vuong et al. (2023)
Individual dimension			
Risk perception	RP_1	Perceived accident risk associated with speeding	He et al. (2021); Vuong et al. (2023)
	RP_2	Perceived accident risk associated with physical fatigue	He et al. (2021)
	RP_3	Perceived accident risk associated with risky riding/maneuvering	He et al. (2021); Vuong et al. (2023)
	RP_4	Overall perceived vulnerability and consequences of motorcycle riding risk	He et al. (2021); Vuong et al. (2023)
Personal traits	PT_1	Anxiety/tendency to worry	McCrae and John (1992); Vuong et al. (2023)
	PT_2	Emotional instability/reactivity	McCrae and John (1992); Vuong et al. (2023)
	PT_3	Anger and irritability	White and Dahlen (2001); Zhang et al. (2017); Vuong et al. (2023)
Work condition dimension			
Work load	WL_1	Mental demand	Hart and Staveland (1988)
	WL_2	Physical demand	Hart and Staveland (1988)
	WL_3	Temporal demand/time pressure	Hart and Staveland (1988)
	WL_4	Overall effort/performance/frustration associated with workload	Hart and Staveland (1988)
Behavior dimension			
Risk behavior	RB_1	Traffic-rule violations	Vuong et al. (2023); He et al. (2021); Nguyen-Phuoc et al. (2024)
	RB_2	Speeding and risky riding/maneuvering violations	Chen and Chen (2011); Vuong et al. (2023)
	RB_3	Other safety-related/aggressive riding violations	Vuong et al. (2023); Zhang et al. (2017);

**Table 2:** Characteristics of Commercial Riding.

Characteristic	Whole Sample (n = 1251)	≤5 Years (n = 799)	>5 Years (n = 452)	*p*-Value
Age (years)	43.5 ± 10.5	41.9 ± 10.6	46.4 ± 9.8	<0.001 ***
Driving experience (years)	17.2 ± 9.6	15.6 ± 9.2	20.0 ± 9.7	<0.001 ***
Household size (persons)	3.0 ± 1.4	3.0 ± 1.4	3.1 ± 1.4	0.172
Driving hours per day	10.7 ± 2.8	10.7 ± 2.8	10.7 ± 3.0	0.842
Longest continuous work (hours)	12.3 ± 2.8	12.3 ± 2.7	12.2 ± 2.9	0.481
Trips per day	24.1 ± 11.0	23.2 ± 10.3	25.7 ± 11.9	<0.001 ***
Monthly riding income (baht)	13,588 ± 6581	13,398 ± 5382	13,925 ± 8286	0.176

Note: Values are presented as mean ± SD and *** indicates *p* ≤ 0.001.

**Table 3:** Variables information.

Variables	Work Experiences								Sig.
	Up to 5 Years				More Than 5 Years				
	Mean	SD	Skewness	Kurtosis	Mean	SD	Skewness	Kurtosis	
Attitude	3.19	0.92	−0.11–−0.09	−0.46–−0.28	3.19	1.04	−0.19–−0.16	−0.74–−0.67	0.929
Subjective norm	2.45	0.89	0.29–0.44	−0.83–−0.53	2.49	0.98	0.46–0.64	−0.89–−0.28	0.529
Perceived behavior control	2.97	0.88	−0.23–0.09	−0.60–−0.36	3.14	0.94	−0.12–−0.01	−0.70–−0.58	0.002 **
Risk perception	3.50	0.78	−0.41–−0.29	−0.52–−0.17	3.65	0.84	−0.61–−0.23	−0.68–−0.04	0.003 **
Personal trait	2.00	0.87	0.88–1.02	0.39–0.48	1.86	0.83	0.98–1.14	0.38–1.01	0.008 **
Workload	12.45	3.83	−0.64–−0.38	−0.05–−0.30	11.61	4.26	−0.48–−0.20	−0.69–−0.22	0.000 ***
Risk behavior	1.97	1.00	0.78–1.25	−0.36–−0.56	1.93	0.84	0.77–1.41	−0.22–−0.95	0.451

Remarks: *** indicates *p* ≤ 0.001, ** indicates *p* ≤ 0.01.

**Table 4:** Measurement Models.

Variables		Model 1				Model 2.1				Model 2.2			
		Loading	CR	α	AVE	Loading	CR	α	AVE	Loading	CR	α	AVE
Attitude	AT_1	0.806	0.701	0.695	0.543	0.844	0.697	0.677	0.541	0.854	0.732	0.722	0.581
	AT_2	0.660				0.607				0.662			
Subjective norm	SN_1	0.697	0.735	0.734	0.480	0.700	0.734	0.734	0.479	0.685	0.738	0.732	0.481
	SN_2	0.720				0.710				0.747			
	SN_3	0.665				0.669				0.653			
Perceived behavior control	PBC_1	0.709	0.743	0.743	0.491	0.679	0.735	0.734	0.481	0.758	0.750	0.749	0.504
	PBC_2	0.733				0.732				0.723			
	PBC_3	0.661				0.667				0.636			
Risk perception	RP_1	0.649	0.775	0.772	0.461	0.608	0.751	0.748	0.428	0.706	0.803	0.799	0.505
	RP_2	0.727				0.691				0.776			
	RP_3	0.682				0.664				0.701			
	RP_4	0.656				0.658				0.646			
Personal trait	PT_1	0.771	0.816	0.814	0.596	0.753	0.822	0.816	0.604	0.808	0.802	0.803	0.577
	PT_2	0.800				0.838				0.720			
	PT_3	0.740				0.732				0.747			
Workload	WK_1	0.682	0.803	0.802	0.507	0.696	0.793	0.793	0.493	0.658	0.813	0.809	0.519
	WK_2	0.800				0.783				0.812			
	WK_3	0.706				0.672				0.746			
	WK_4	0.656				0.648				0.666			
Risk behavior	RB_1	0.735	0.760	0.757	0.512	0.784	0.798	0.792	0.565	0.608	0.665	0.668	0.408
	RB_2	0.710				0.775				0.567			
	RB_3	0.703				0.698				0.719			

**Table 5:** Correlation Matrix of the Research Variables.

	AT	SN	PBC	RP	PT	WK	RB
AT	1						
SN	0.067–0.087	1					
PBC	0.099–0.158	0.147–0.238	1				
RP	0.434–0.474	0.126–0.204	0.101–0.172	1			
PT	0.046–0.107	0.425–0.522	0.249–0.280	0.270–0.342	1		
WK	0.055–0.106	0.138–0.155	0.049–0.131	0.171–0.364	0.063–0.076	1	
RB	0.096–0.289	0.312–0.440	0.074–0.194	0.161–0.200	0.432–0.473	0.066–0.073	1

Note: HTMT = Heterotrait–Monotrait ratio. Values presented as ranges represent the lower and upper bounds of the bootstrapped confidence interval for each HTMT estimate.

**Table 6:** Summary of Hypothesis Testing Results.

Hypothesis	Proposed Effect	Model 1	Model 2.1	Model 2.2	Conclusion
		(Overall Result)	(≤5 Years)	(>5 Years)	
H1: Attitude → risky violation behavior	Negative	β = −0.112,	Not significant	β = −0.196,	Supported
		significant		significant	
H2: Subjective norm → risky violation behavior	Positive	β = 0.253,	β = 0.288,	β = 0.192,	Supported
		significant	significant	significant	
H3: Perceived behavioral control → risky violation behavior	Positive	Not significant	Not significant	Not significant	Not supported
H4: Risk perception → risky violation behavior	Negative	Not significant	Not significant	Not significant	Not supported
H5: Personal traits → risky violation behavior	Positive	β = 0.330,	β = 0.289,	β = 0.396,	Supported
		significant	significant	significant	
H6: Workload → risky violation behavior	Positive	β = −0.096,	β = −0.125,	Not significant	Not supported
		significant	significant		

## Data Availability

The data are not publicly available due to ethical reasons and privacy considerations.
